# Present and Possible Therapies for Age-Related Macular Degeneration

**DOI:** 10.1155/2014/608390

**Published:** 2014-04-16

**Authors:** Muhammad Khan, Ketan Agarwal, Mohamed Loutfi, Ahmed Kamal

**Affiliations:** ^1^School of Medicine, University of Liverpool, Liverpool L69 3GE, UK; ^2^Ophthalmology Department, University Hospital Aintree, Liverpool L9 7AL, UK

## Abstract

Age-related macular degeneration (AMD) is the most common cause of blindness in the elderly population worldwide and is defined as a chronic, progressive disorder characterized by changes occurring within the macula reflective of the ageing process. At present, the prevalence of AMD is currently rising and is estimated to increase by a third by 2020. Although our understanding of the several components underpinning the pathogenesis of this condition has increased significantly, the treatment options for this condition remain substantially limited. In this review, we outline the existing arsenal of therapies available for AMD and discuss the additional role of further novel therapies currently under investigation for this debilitating disease.

## 1. Introduction

The concept of vision has been considered an enigma that has tantalised and tested eminent scholars since antiquity. Although great strides have been made in our understanding of the mechanisms underpinning vision, for most individuals, the ability to see is often underappreciated on a daily basis as it is deemed integral and innate to their livelihood. However, perceiving a life wherein vision was just an abstract concept and could be merely described but not experienced. For over 39 million individuals, this is their reality as they must face the ramifications associated with their blindness both physically and psychologically. Despite there being several causes of visual impairment and blindness, one deemed the most notorious is age-related macular degeneration (AMD) [1].

AMD accounts for the leading cause of blindness in those aged ≥55 [2], in addition to underpinning two-thirds of all registrations of visual impairment/blindness within the UK. Presently, AMD is defined as changes occurring within the macula reflective of the ageing process that occurs without any obvious precipitating cause [3]. Nevertheless, AMD is an umbrella term that encompasses two pathologically overlapping, yet distinct, processes: geographic atrophy (GA) (dry) AMD and neovascular (wet) AMD [4].

Clinically, the presentation of AMD differs depending upon the development of neovascular or GA AMD. With regard to GA, diagnosis is often incidental due to its insidious nature [5]. However, as the disease progresses, patients often characteristically report difficulties with reading small sized font which escalates to encompass larger sized fonts. In contrast to this, neovascular AMD is characterised by symptoms encompassing visual blurring and distortion within the central field of vision. In addition to this, patients often report a phenomenon known as metamorphopsia, whereby straight lines appear either crooked or wavy. In individuals where neovascular AMD affects one eye only, they often report being oblivious to the aforementioned signs and symptoms. Nevertheless, when bilateral involvement occurs, patients state an acute loss in visual ability, thereby rendering them incapable of reading, driving, or distinguishing facial features and expressions [4]. Unfortunately, both GA and neovascular AMD orchestrate a progressive and unremitting sequential loss of central vision within the affected eye(s) cumulating to blindness.

Understanding the implications of AMD, significant research has been conducted on identifying risk factors for AMD. Several risk factors have been noted to increase the likelihood of developing AMD, yet, by definition, the most significant is an increasing age [4]. Incorporating this realisation alongside an ageing elderly population worldwide, epidemiologists predict that the prevalence of AMD will increase by a third by 2020 [6]. Furthermore, with economic costs attributed to visual impairment secondary to AMD being an estimated $575 to 733 million dollars, the estimated rise in the prevalence of AMD will evidently impose a significant burden on global healthcare systems already under turmoil due to the economic recession [7].

In light of this, substantial investments have been made into dampening the consequences of this debilitating disease. With an estimated worth of four billion US dollars a year, the market for AMD treatments provides a lucrative niche that serves as a “carrot on a stick” for pharmaceutical companies [8]. Presently, significant developments have been made with regard to the therapeutic options available for AMD. This review aims to provide an overview on both the current and emerging interventions which may serve as the future treatments for AMD. However, prior to doing so, it is imperative to provide a background on the pathogenesis of AMD.

## 2. Pathogenesis of AMD

Our current understanding behind the pathogenesis of AMD stipulates that there is no predominant aetiological factor dictating the development of AMD. Rather, there is a multifactorial element to AMD, whereby interactions between several facets intertwine and coordinate a cascade of sequential steps that provide the appropriate environment for AMD to flourish [9]. However, implicated for both forms of AMD are the involvement and degeneration of four principle ocular regions: the outer retina, the retinal pigment epithelium (RPE), Bruch's membrane (BM), and the choriocapillaris [10]. Although the intricate processes explaining their degeneration still remain elusive, four mechanisms have been postulated as being imperative to the formation of AMD: lipofuscinogenesis, drusogenesis, inflammation, and choroidal neovascularisation; the former three aspects are critical to formation of both types of AMD, whereas the last represents the final stage in the development of neovascular AMD [11, 12].

### 2.1. Lipofuscinogenesis

Within the outer retina resides a monolayer of postmitotic cells referred to as the RPE. Regarded as the mediator of retinal homeostasis, the RPE plays a vital role in the maintenance of retinal photoreceptors [13]. However, over the course of senescence, there is progressive dysfunction of the RPE, thereby inducing a state of metabolic insufficiency which results in the formation and accumulation of lipofuscin. Deemed highly potent, due to the major component of lipofuscin being N-retinylidene-N-retinyl ethanolamine (A2E), the A2E produced has the ability to interfere with the functional aspects of the RPE, thus triggering apoptosis of the RPE with subsequent development of GA [10, 13]. Furthermore, the accumulation of A2E within the RPE has been shown to increase the risk of choroidal neovascularisation and so neovascular AMD [14].

### 2.2. Drusogenesis and Inflammation

Pathognomonic in the development of AMD is the formation of drusen. Defined as “discrete lesions consisting of lipids and proteins” [15], these amorphous deposits accumulate within the region situated between the RPE and the BM. Depending upon their size and shape, drusen are clinically categorised into either small (diameter ≤ 63 *μ*m)/large (diameter ≥ 125 *μ*m) “soft” drusen or small/large (definitions identical to before) “hard” drusen [16]. Their clinical significance differs as relatively few quantities of small, hard drusen have been identified in over 95% of the elderly population and are regarded as a benign occurrence. Nevertheless, presence of large, hard and/or large, soft drusen has been recognised as increasing the risk of AMD. One component of this affiliation orientates around the physical displacement, and resulting death, of clusters of photoreceptors within the RPE overlying the drusen, thus leading to GA AMD [17]. In addition to this, formation of soft drusen is associated with the detachment of the RPE from the BM, thereby incurring extensive damage to the principle ocular regions and inducing the development of neovascular AMD [10].

Another dimension to the relationship between drusogenesis and AMD occurs through the indirect influence of drusen on the immune system [18]. Indeed, identification of several components of the immune system, such as macrophages, complement component 3 (C3), and the membrane attack complex (MAC), within drusen has raised the possibility that drusen mediated inflammation, with subsequent activation of the complement cascade, may lead to notable degeneration and disruption to both the RPE and BM by autologous tissue/cell damage by the MAC [18, 19].

### 2.3. Choroidal Angiogenesis

There is a delicate balance within endothelial cells residing in the retinal vasculature between factors that promote angiogenesis, such as vascular endothelial growth factor (VEGF), and those that inhibit it. In fact, it is the maintenance of this homeostatic mechanism that ensures negligible proliferation of endothelial cells with ensuing neovascularisation. However, in neovascular AMD, there is a pathological shift in favour of factors promoting angiogenesis [20]. With regard to the causative factor for this shift, it is postulated that the inflammation and recruitment of several components of the immune system trigger the release of proangiogenic mediators such as VEGF, thereby forming a milieu that favours angiogenesis [21]. Regardless of the exact mechanism, progression to neovascularisation leads to the formation and extension of permeable, weak, and leaky vessels from the vascular choriocapillaris to the avascular choroid which, in turn, induces local oedema but, more profoundly, acute central vision loss resulting from haemorrhage with successive development of a fibrous scar (disciform scar) [10].

## 3. Preventative Measures for GA and Neovascular AMD

Patients are initially subject to extensive risk stratification, whereby modifiable risk factors are either reduced or nullified. At present, the risk factors demonstrated to be efficacious following their minimisation are smoking and diets low in antioxidants. The casual relationship between smoking and AMD has been noted to significantly increase the risk of developing AMD by two- to threefold; therefore smoking cessation is heavily emphasised [22]. Furthermore, patients are advised to have a diet rich in foods with antioxidant micronutrients. As the retina is highly susceptible to oxidative stress from the effects of visible light and production of oxygen radicals, as denoted by the “free radical” theory of ageing, then diets low in antioxidants would only augment this process [23]. Several studies have examined whether consumption of high amounts of antioxidant micronutrients within a diet would serve as a protective role for the progression and development of AMD. Although a recent Cochrane review showed no evidence for the role of antioxidant supplementation in the prevention of AMD [24], the Age-Related Eye Disease Study (AREDS) noted a markedly reduced risk of progression of AMD if individuals consumed antioxidants such as zinc, *β*-carotene, vitamin C, and vitamin E [16]. However, due to *β*-carotene increasing the risk of respiratory malignancy in smokers, the AREDS2 study revised the formulation by replacing *β*-carotene with the carotenoids, lutein and zeaxanthin [25].

Although preventative measures serve as one arm in the holistic management of AMD, they only serve as adjuncts to the treatments described below.

## 4. Present Treatments for Neovascular AMD

### 4.1. Anti-VEGF Therapies

Revolutionising the management of neovascular AMD, anti-VEGF therapies are regarded as the “gold-standard” treatment. Aiming to restore the angiogenic imbalance, anti-VEGF therapies unequivocally not only reduce the exudative changes but also provide significant improvements in visual function. Currently, the following three anti-VEGF therapies are used for the treatment of neovascular AMD: ranibizumab (Lucentis), bevacizumab (Avastin), and aflibercept (Eylea) [26, 27].

A humanised Fab fragment of IgG1-*κ* monoclonal antibody, ranibizumab, binds to and inhibits all isoforms of VEGF-A; VEGF-A is a gene that belongs to the VEGF family and codes for a protein implicated in angiogenesis [28]. Despite proven to be efficacious [29, 30], concerns have been raised regarding the safety of this drug due to the implications of VEGF in thrombus formation and development of atherosclerotic plaques [28]. To address these concerns, studies were conducted to identify the risk of exposure to cardiovascular events such as thrombosis, hypertension, and haemorrhage. Despite both studies incorporating patients with a prior history of cardiovascular disorders and concluding no significant difference in the incidence of such adverse events, their findings were deemed inconclusive due to their insufficient sample sizes [29, 30]. Alongside this, the safety concerns regarding this anti-VEGF agent have been augmented following studies noting that it may potentially increase the risk of stroke [31].

Bevacizumab is also a humanised IgG1 monoclonal antibody, yet it differs from ranibizumab, as it represents the entire molecule and not simply a Fab fragment [28]. In addition, it is less affinity matured than ranibizumab but binds to more isoforms of VEGF [28]. Through large clinical trials, bevacizumab has demonstrated similar efficacy and safety results as ranibizumab [32, 33]. However, due to the lack of long-term trials demonstrating its safety, bevacizumab is currently restricted to off-label use only [27].

Acting as a fusion protein, aflibercept specifically binds to all isoforms of VEGF-A [28]. Due to its ability to penetrate further within the retina and bind with a greater affinity than existing treatments, aflibercept demonstrates the same efficacy as ranibizumab but, additionally, requires fewer subsequent intravitreal injections than ranibizumab [34, 35]. Aflibercept has recently undergone appraisal by the National Institute for Health and Care Excellence (NICE) and is now licensed for its use in neovascular AMD [36].

## 5. Future Treatments for Neovascular AMD

Although anti-VEGF treatments represent the mainstay of treatment, the progressive decline in their biological efficacy, as a result of tachyphylaxis, is quite concerning as they may only be beneficial on a short-term basis with no long-term efficacy. [37]. Furthermore, a significant limitation of such therapies is that they are delivered on a repeated basis through intravitreal injections. Therefore, they expose the patient to substantial side effects such as retinal detachment, endophthalmitis, and traumatic cataract [32]. Most significantly however, is whether existing treatments for neovascular AMD are simply a palliative measure or do they actually provide a cure AMD?

Despite such interventions aiming to inhibit further pathological angiogenesis, some studies have demonstrated that they play no active role in reestablishing an optimum interface between the principle ocular regions required for central vision [38]. Rather, such treatments, at best, lead to the formation of a disciform scar which only augments the loss of vision due to its physical presence disrupting the above interface. In addition to this, present treatments only provide therapeutic benefit to those with the active form of neovascular AMD, therefore being futile and redundant for those who have already lost their vision [38]. With such significant limits, further research is being conducted on novel options which address the aforementioned issues.

### 5.1. Combination Therapies

To minimise the risks associated with intravitreal injections, attention has shifted into agents that reduce the frequency of injections required without compromising on their efficacy. One method of achieving this is to combine existing anti-VEGF therapies with other treatment modalities. The first example of this was the combination of ranibizumab with photodynamic therapy (PDT); PDT refers to the intravenous injection of the photosensitive drug verteporfin which, upon activation by laser light, induces injury to endothelial cells within retinal vasculature with subsequent thrombosis of said vessels [38]. Although promising, a collation of results from several studies, deemed as the SUMMIT trials, have shown that combination therapy provided no significant reduction in the frequency of injections required [28]. Presently, attention has shifted to the role of a concoction of PDT, an anti-VEGF, and a corticosteroid such as dexamethasone (triple therapy). The rationale behind this regimen is in three parts with an initial eradication of existing neovascular AMD following PDT, the corticosteroid dampening the inflammatory response, and, lastly, inhibition of angiogenesis by the anti-VEGF [28]. Currently, the literature regarding the efficacy of this regimen is sparse; however, a Phase II study, known as the RADICAL study, identified an improvement in visual acuity and a statistically significant reduction in the number of retreatments in the cohort receiving half-fluence PDT, dexamethasone, and ranibizumab compared to ranibizumab alone [39]. With such promising results, the use of triple regimen may provide one means of alleviating the toll of frequent injections whilst ensuring improvements in vision.

Alongside combining several treatment modalities, investigation is also underway into alternative therapies that act on various components in the pathogenesis of neovascular AMD.

### 5.2. VEGF Signalling Inhibitors

Interventions targeting VEGF continue to be at the forefront of research. However, focus has shifted to the inhibition of components implicated in the angiogenic signalling cascade leading to the formation of VEGF. One example of this is to utilise tyrosine kinase inhibitors which disrupt the downstream signalling of VEGF by inhibiting the phosphorylation of the tyrosine residue of the kinase domain present on VEGF receptors (VEGFRs), of which there are three forms: VEGFR1, VEGFR2, and VEGFR3, thereby preventing both the activation of these receptors and their subsequent angiogenic effects [40]. Presently, several different tyrosine kinase inhibitors are in various stages of clinical trials. Pazopanib is a tyrosine kinase inhibitor that selectively inhibits all the three forms of the VEGFR receptor, in addition to inhibiting other factors implicated in angiogenesis such as platelet-derived growth factor receptor (PDGFR) and c-KIT [40]. Results from a Phase II study have identified its potential role in treatment of neovascular AMD with particular emphasis given to its application via topical eye drops providing a means of alleviating any potential side effects associated with present intravitreal injections [41]. In a similar manner to pazopanib, vatalanib acts as a tyrosine kinase inhibitor with a specific affinity for all subtypes of the VEGFR and yet differs as it is given orally [40]. Although it has undergone both Phase I and Phase II studies, results are still yet to be published regarding whether this agent is efficacious against neovascular AMD. TG100801 is another example of a tyrosine kinase inhibitor that demonstrates an affinity for inhibiting all subtypes of the VEGFR and PDGFR. Despite its application in a Phase I trial being successful, its progression to a Phase II trial was abruptly terminated due to developments of corneal toxicity [40].

### 5.3. Gene Therapy

Gene transfer provides a very promising prospect where a single injection could possibly replace the repeated injection regimen [42]. By augmenting the expression of endogenous proteins (i.e., inhibitors of VEGF, endostatin, or pigment epithelium-derived factor) through intravitreal or subretinal injection of viral vectors, scientists have shown a reduction in neovascularisation in animal models and early clinical trials [42]. However, the study of this therapy is still in its infancy with serious toxicity matters to overcome [42].

### 5.4. Integrin Antagonists

Integrins refer to transmembrane proteins which are a pivotal aspect of angiogenesis as they mediate the migration of endothelial cells. By inhibiting the effect of integrins, it is postulated that angiogenesis will be inhibited. At present, emphasis is primarily on the integrin *α*5*β*1, which is known to be expressed on the surface of endothelial cells found within the vasculature [40]. Currently, Phase I trials are being conducted into agents that antagonise the effects of integrin such as the direct antagonist JM6427 (ClinicalTrials.gov identifier: NCT00536016) and the monoclonal antibody volociximab (ClinicalTrials.gov identifier: NCT00782093).

### 5.5. Mammalian Target of Rapamycin (mTOR) Inhibitors

mTOR refers to a tyrosine kinase which is involved in the regulation of cell growth and proliferation. Following its activation, mTOR induces the production of hypoxia-inducible factors (HIF), such as HIF-1a, which subsequently triggers the expression of VEGF [40]. Although several mTOR inhibitors are undergoing clinical trials, the mTOR inhibitor everolimus is the only class of this drug undergoing a Phase II clinical trial related to its efficacy in neovascular AMD (ClinicalTrials.gov identifier: NCT00304954).

### 5.6. Inhibition of the Complement Pathway

As discussed before, the complement cascade has a pivotal role in the pathogenesis of AMD. POT-4 is a synthetic peptide that reversibly binds to C3 and inhibits it [40]. Results from a Phase I study, known as the ASaP trial, revealed that the use of POT-4 in patients with neovascular AMD noted no safety concerns (ClinicalTrials.gov identifier: NCT00473928). Currently, Phase II trials are being planned to determine its efficacy and safety when used alongside ranibizumab [40].

Furthermore, although the use of ARC-1905 was previously discussed with its potential role as a treatment for GA AMD, results from a concurrent Phase I trial have yet to be published where the tolerability and safety of ARC-1905, in combination with ranibizumab, were determined for neovascular AMD (ClinicalTrials.gov identifier: NCT00709527).

Alongside research on novel pharmacotherapies, other modalities such as radiotherapy and maculoplasty are now being explored with the aim of identifying other therapies for neovascular AMD.

### 5.7. Radiotherapy

Although radiotherapy alone has not shown to offer any significant improvements in visual acuity, the role of radiotherapy as an adjunct therapy has become a topic of recent interest [43]. Initial trials such as the MERITAGE study concluded that epiretinal brachytherapy not only produced an improvement in visual acuity but also reduced the need for subsequent retreatment with anti-VEGF therapy [44]. Nevertheless, a more recent trial known as the CABERNET trial failed to reproduce these findings, thus raising speculation as to the true role of radiotherapy in the management of neovascular AMD [45]. However, attention has shifted into exploring other uses of radiotherapy such as the role of radiotherapy as an adjunct to patients who fail to respond to anti-VEGF therapy which is currently under investigation in the Phase IV MERLOT trial (ClinicalTrials.gov identifier: NCT01006538). Furthermore, studies such as the INTREPID study have identified that the use of the iRAY device, a device that allows for delivery of high doses of radiation without requiring an invasive procedure, in sufferers of neovascular AMD, being managed with anti-VEGF therapy alone, resulted in a significant reduction of the frequency of retreatments required with no serious adverse events being recorded [46].

### 5.8. Maculoplasty

Regarded as a means of reconstructing the subretinal architecture, various surgical modalities have been devised for both GA and neovascular AMD which are encompassed within the term maculoplasty. Integral to such surgical techniques is the principle aim of ameliorating, and not simply dampening the progression of central vision by restoring the interface between the outer portion of the retina, RPE, BM, and the choriocapillaris [11]. Although this has been attempted previously, through surgeries either inserting a graft segment of free peripheral RPE-choroid under the existing fovea or relocating healthy photoreceptors within the fovea to adjacent areas of healthy intact RPE, they were deemed inappropriate due to their significant surgical complications despite both stabilising and improving near and distance vision [47]. Nevertheless, preliminary studies are now being conducted into further techniques such as transplantation of the RPE [48], repopulation of the RPE in an aged BM through RPE grafts [49], and restoration of the RPE through use of stem cells [50].

Although being in its infancy, further refinement of maculoplasty techniques may pave the way for developing a treatment modality which is not only efficacious and safe, but also more importantly provides the first intervention which restores vision in patients suffering from both forms of AMD.

## 6. Present and Future Treatments for GA AMD

Despite not being exhaustive, current treatments for neovascular AMD provide some therapeutic options of known efficacy. However, with GA AMD, there are currently none whatsoever. However, two principle strategies are presently being explored to prevent the decline in central vision from GA AMD: photoreceptor and RPE preservation and inhibition of the complement cascade [51].

### 6.1. Photoreceptor and RPE Preservation

One method of ensuring preservation of both the photoreceptors and the RPE is to devise agents that ensure adequate circulation to the choroidal vasculature, thereby preventing apoptosis secondary to ischaemia. Exhibiting cytoprotective effects in areas of ischaemia [51], trimetazidine was shown in trials to be a preventative therapy for GA AMD [52]. Another example of a drug acting on this mechanism is MC-1101 which not only increases vascular supply to the choroid but also, furthermore, exhibits both antioxidant and anti-inflammatory properties, both of which are implicated in the pathogenesis of AMD [51]. A Phase II/III trial has recently been initiated which is set to complete in October 2014 (ClinicalTrials.gov identifier: NCT01601483).

Another tact adopted is to utilise neuroprotective agents such as the cytokine ciliary neurotrophic factor (CNTF), which has been shown to inhibit the apoptosis of photoreceptors [40]. With such promising potential, a sustained release device known as NT-501 was devised that allowed for the passage of CNTF from the device to the region of interest. At present, results from a Phase II trial which investigated the improvement in visual acuity following use of CNTF via this device have yet to be published (ClinicalTrials.gov identifier: NCT00447954).

As stated before, accumulation of A2E plays an integral role in the development of GA AMD [10, 13]. With this in mind, research has been conducted on drugs that reduce the formation of A2E. Fenretinide is one example of these and has shown to halt the formation of A2E [40]. Recent results from a Phase II trial noted that fenretinide over a two-year period was safe but, furthermore, reduced the rate of GA enlargement in patients with GA AMD [53]. Another example of a drug targeting this mechanism is ACU-4429. By inhibiting the isomerisation of all-*trans*-retinyl ester to 11-*cis*-retinol, ACU-4429 effectively reduces the rate of the visual cycle and so the accumulation of A2E. Following reports from Phase I trials demonstrating its safety and tolerability in healthy patients [54], a Phase II/III trial commenced in February 2013 with the aim of determining its efficacy in reducing the progression of GA AMD (ClinicalTrials.gov identifier: NCT01002950).

### 6.2. Inhibition of the Complement Cascade

As an inhibitor of the cleavage of C5 to C5a and C5b, the monoclonal antibody eculizumab prevents the downstream activation of the potent MAC [40]. Its efficacy and safety in the treatment of GA AMD were recently investigated in the ongoing Phase II trial known as the COMPLETE study which aimed to determine the efficacy of eculizumab in reducing the progression of GA AMD (ClinicalTrials.gov identifier: NCT00935883). Similarly, the pegylated aptamer ARC-1905 also inhibits the activation of the MAC by blocking the cleavage of C5 [40]. Currently, a Phase I study is determining the role of this agent in GA AMD (ClinicalTrials.gov identifier: NCT00950638).

In contrast to the above, lampalizumab (FCFD4514S) is a monoclonal antibody Fab fragment that inhibits complement factor D which is the rate limiting enzyme in the alternative pathway of the complement cascade. Through inhibition, researchers hope to attenuate the downstream components of the complement cascade, thus reducing the effects of the MAC [51]. A recent Phase II trial demonstrated a good safety profile for the drug and a statistically significant reduction in geographical atrophy in patients treated with monthly intravitreal injections of lampalizumab over an 18-month period providing promising results for slowing down of the atrophy process [55].

## 7. Conclusion

Over the last decade the way we have perceived and understood AMD has dramatically changed. As we continue to increase our knowledge regarding the intricate mechanisms underpinning this debilitating disease, several further treatment options are now being pursued in the hope of increasing the armamentarium available for the treatment of AMD. However, one avenue which could completely transform the field is the role of maculoplasty. Integrating these various aspects of AMD, there may indeed be light at the end of the tunnel for both researchers and patient's alike as, with further research, we may be on the cusp of devising a means of effectively halting the progression of AMD, but, furthermore, we may be on the verge of finding a cure for AMD.
